# A preliminary study of the effect of closed incision management with negative pressure wound therapy over high-risk incisions

**DOI:** 10.1186/s12917-015-0593-4

**Published:** 2015-11-09

**Authors:** Karen L. Perry, Lynda Rutherford, David M. R. Sajik, Mieghan Bruce

**Affiliations:** Department of Small Animal Clinical Sciences, College of Veterinary Medicine, Michigan State University, 736 Wilson Road, East Lansing, MI 48824 USA; Department of Clinical Science and Services, The Royal Veterinary College, Hawkshead Lane, North Mymms, Hatfield, Hertfordshire AL9 7TA UK; Department of Production and Population Health, The Royal Veterinary College, Hawkshead Lane, North Mymms, Hatfield, Hertfordshire AL9 7TA UK

**Keywords:** Negative Pressure Wound Therapy, High-Energy Fracture, Arthrodesis, Swelling, Wound discharge

## Abstract

**Background:**

Certain postoperative wounds are recognised to be associated with more complications than others and may be termed high-risk. Wound healing can be particularly challenging following high-energy trauma where wound necrosis and infection rates are high. Surgical incision for joint arthrodesis can also be considered high-risk as it requires extensive and invasive surgery and postoperative distal limb swelling and wound dehiscence are common. Recent human literature has investigated the use of negative pressure wound therapy (NPWT) over high-risk closed surgical incisions and beneficial effects have been noted including decreased drainage, decreased dehiscence and decreased infection rates. In a randomised, controlled study twenty cases undergoing distal limb high-energy fracture stabilisation or arthrodesis were randomised to NPWT or control groups. All cases had a modified Robert-Jones dressing applied for 72 h postoperatively and NPWT was applied for 24 h in the NPWT group. Morphometric assessment of limb circumference was performed at six sites preoperatively, 24 and 72 h postoperatively. Wound discharge was assessed at 24 and 72 h. Postoperative analgesia protocol was standardised and a Glasgow Composite Measure Pain Score (GCPS) carried out at 24, 48 and 72 h. Complications were noted and differences between groups were assessed.

**Results:**

Percentage change in limb circumference between preoperative and 24 and 72 h postoperative measurements was significantly less at all sites for the NPWT group with exception of the joint proximal to the surgical site and the centre of the operated bone at 72 h. Median discharge score was lower in the NPWT group than the control group at 24 h. No significant differences in GCPS or complication rates were noted.

**Conclusions:**

Digital swelling and wound discharge were reduced when NPWT was employed for closed incision management. Larger studies are required to evaluate whether this will result in reduced discomfort and complication rates postoperatively.

## Background

Certain postoperative wounds are recognised to be associated with more complications than others and may be termed high-risk. Problems associated with wound healing may lead to postoperative wound dehiscence, infection and additional surgical procedures [1]. Wound healing can be particularly challenging following high-energy trauma to the soft tissue and underlying bone, such as with high-energy fractures, with wound necrosis and infection rates reaching as high as 33–50 % in some series [1]. Surgical incisions following joint arthrodesis can be considered high-risk as arthrodesis is an extensive and invasive surgery, involving more soft tissue trauma and subsequent inflammation than typical internal fixation [2], with significant postoperative swelling and compromised circulatory drainage of the distal limb being possible sequelae [2]. Distal limb swelling features significantly as a postoperative complication following arthrodesis as this can result in difficultly achieving a tension free closure and predispose to postoperative infection [3, 4]. In some cases severe wound dehiscence following arthrodesis has been reported to necessitate euthanasia or limb amputation [5].

The promotion of wound healing through localised suction drainage is accepted practice [6, 7]. It has been suggested that components of excess wound fluid may serve as both physical and chemical deterrents to wound healing [7]. The positive manipulation of tissue growth by applied mechanical forces is also well established, as in distraction osteogenesis and soft tissue expansion. Negative pressure wound therapy (NPWT) achieves both with the primary mechanisms of action being removal of excess wound fluid and transmission of mechanical forces to the surrounding tissue with resultant deformation of the extracellular matrix and cells [8]. The technique of NPWT involves the use of an open-cell foam or gauze dressing placed in the wound defect and sealed with an adhesive drape. A fenestrated evacuation tube is connected to an adjustable vacuum source, applying controlled NPWT uniformly to all tissues on the inner surface of the wound [9, 10]. This technique converts an open wound into a controlled and temporarily closed environment [10], and can be used to achieve a variety of treatment goals which vary according to the patient and wound characteristics [11]. The advantage of this is to increase and facilitate wound healing. It has become a favoured method for wound management in human patients because of its simple nature and ability to manage complex wounds with high efficacy [9].

Numerous applications of NPWT have been reported in humans including wounds with exposed bone, tendon or hardware, acute burns and as an adjunct to skin grafting [9]. In the veterinary literature, reported uses have included acute open wounds [12], a large skin wound over the dorsum of a cat [13], distal extremity wounds [14], peristomal necrosis following prepubic urethrostomy [15], urine-induced skin and thigh muscle necrosis following traumatic urethral rupture [16], wounds with exposed metallic implants [17] and as an adjunct to free full-thickness skin grafts [8]. Negative pressure wound therapy has also been found to be a useful adjunct in the treatment of open high-energy injuries in human orthopaedic patients [18]. Most current studies have focused on the use of NPWT in open wounds to assist healing by secondary intention, however, based on the mechanisms of action described, the favourable effects of NPWT should exist with a surgical wound that has been sutured but which is draining [1]. Recent human literature has investigated the use of NPWT over high-risk closed surgical incisions such as following tibial plateau, pilon and calcaneal fractures [19] and median sternotomy [20] wounds which are associated with a high risk of complications including dehiscence. Beneficial effects have been cited as decreased drainage, decreased incidence of dehiscence, decreased infection rates and improved wound healing [1, 19, 20]. A further study using finite element analysis and bench modelling, demonstrated that the application of NPWT over closed surgical incisions decreased the lateral stresses around the incision, changed the direction of the stresses to a distribution more typical of intact tissue and increased the force required to disrupt the closed incision by approximately 50 % [21]. It is also postulated that this adjunct to wound closure may decrease the pain and swelling associated with these wounds [22].

The authors have experienced a high rate of distal limb swelling and wound complications following high-energy fractures or arthrodesis of the distal limbs, particularly in feline patients. The purpose of our study was to evaluate the use of NPWT over these high-risk incisions by assessing limb swelling, wound drainage, pain and complication rate with patients managed with NPWT compared to standard postoperative management. A further objective of this pilot study was to obtain data which would allow sample sizes to be calculated for a further study designed to demonstrate a significant effect. Our hypothesis was that the use of NPWT would significantly reduce distal limb swelling, wound drainage, pain and complication rate when applied over these high-risk incisions.

## Results

Twenty animals were enrolled in the study, 13 (65 %) dogs and 7 (35 %) cats which were divided into ten NWPT and ten control patients. Demographic data are summarised in Table 1. No data was missing except for long-term complications in two of the control group and two NPWT patients that were lost to follow-up (duration of follow-up for these patients ranged from 3–13 days). Patients were followed-up for a median of 10 weeks (range 1 to 40 weeks).

**Table 1:** Demographic data for control and NPWT groups^a^

Variable	Control (*n* = 10)	NPWT (*n* = 10)	*p*-value^b^
Species			1
Canine	7 (70 %)	6 (60 %)	
Feline	3 (30 %)	4 (40 %)	
Age	31.5 (17.50 to 41.25)	83.5 (45.2 to 105.0)	0.03
Sex			0.61
Male entire	4 (40 %)	2 (20 %)	
Male neutered	3 (30 %)	5 (50 %)	
Female entire	1 (10 %)	0 (0 %)	
Female neutered	2 (20 %)	3 (30 %)	
Sex (male or female)			1
Male	7 (70 %)	7 (70 %)	
Female	3 (30 %)	3 (30 %)	
Limb			0.61
Right thoracic	5 (50 %)	3 (30 %)	
Left thoracic	3 (30 %)	3 (30 %)	
Right pelvic	0 (0 %)	2 (20 %)	
Left pelvic	2 (20 %)	2 (20 %)	
Limb (thoracic or pelvic)			0.63
Thoracic	8 (60 %)	6 (60 %)	
Pelvic	2 (20 %)	4 (40 %)	
Surgery type			0.37
Fracture	7 (70 %)	4 (40 %)	
Arthrodesis	3 (30 %)	6 (60 %)	
Duration of follow-up	10.5 (1 to 36)	10.0 (1 to 40)	0.85

^a^Data are median (25^th^ to 75^th^ quartile) or numbers (%) in each group

^b^Assessment of differences between groups (Fisher’s exact test or Mann–Whitney U test)

There were six (30 %) male entire, eight (40 %) male neutered, one (5 %) female entire and five (25 %) female neutered animals in the study. Ages ranged from 9 months to 144 months (12 years), the median age was 43 months (3 years and 7 months).

Eight (40 %) animals had surgery on the right thoracic limb, six (30 %) on the left thoracic limb, two (10 %) on the right pelvic limb and four (20 %) on the left pelvic limb. There were 11 fracture repairs and 9 arthrodesis procedures. Seven (64 %) fracture cases were randomised to the control group and four (36 %) to the NPWT group. Three (33 %) arthrodesis cases were randomised to the control group and six (67 %) to the NPWT group.

There were no significant differences between the treatment groups except for age. The median age in the controls was 31.5 months (IQ: 17.5 to 41.25) compared to 83.5 (IQ: 45.25 to 105.0) months in the NPWT group (Mann–Whitney test *p* = 0.03).

### Percentage change from preoperative measurement comparing control and NPWT groups

The primary outcome of interest was the postoperative swelling of the digits (percentage change from preoperative measurements at the proximal interphalangeal joints (PIPJ) and distal interphalangeal joints (DIPJ). At the PIPJ, the difference in median percentage change from preoperative measurement between the control and NPWT groups was −27.1 % (CI: −38.7 to 11.3 %) at 24 h postoperatively and −24.8 % (CI: −44.7 to −7.7 %) at 72 h. At the DIPJ the difference in the median percentage change between control and NPWT groups was −28.7 % (CI: −37.2 to −15.8 %) at 24 h postoperatively and −26.5 % (CI: −50.0 to −7.0 %) at 72 h postoperatively. The percentage change for the six sites on the limb that underwent surgery at 24 and 72 h postoperatively and the average percentage change at these times are summarised in Table 2. The percentage change was lower in the NPWT group at all sites, at both times, with significant differences observed at all sites except at the joint proximal to the surgical site at 72 h, and the mid-bone measurement at 72 h.

**Table 2:** The median percentage change from preoperative limb circumference in control and NPWT groups and the difference in medians measured 24 and 72 h postoperatively

		Median (25^th^ to 75^th^ Quartile)				Difference in medians (95 % CI)		
Measurement	Time	Control (*n* = 10)		NPWT (*n* = 10)				*p*-value^a^
Proximal interphalangeal joint	24 h	19.1	(10.2 to 24.6)	−8.0	(−14.1 to −0.6)	−27.1	(−38.7 to −11.3)	0.002
	72 h	14.6	(4.7 to 23.2)	−10.2	(−16.7 to 0.8)	−24.8	(−44.7 to −7.7)	0.002
Distal interphalangeal joint	24 h	20.3	(14.0 to 27.0)	−8.2	(−11. to −1.4)	−28.7	(−37.2 to −15.8)	0.0008
	72 h	16.7	(8.6 to 35.0)	−9.8	(−12.0 to 2.7)	−26.5	(−50.0 to −7.0)	0.009
Surgical site	24 h	11.0	(7.2 to 19.0)	3.1	(1.5 to 6.3)	−7.9	(−19.3 to −1.7)	0.02
	72 h	11.6	(6.2 to 18.6)	1.5	(−1.6 to 4.6)	−10.1	(−22.7 to −1.4)	0.04
Joint proximal to surgical site	24 h	8.2	(2.4 to 13.3)	0.0	(−0.6 to 1.0)	−8.2	(−15.2 to −1.1)	0.02
	72 h	5.6	(1.5 to 8.9)	0.0	(−2.0 to 0.9)	−5.6	(−11.4 to −3.6)	0.16
Joint distal to surgical site	24 h	17.7	(10.5 to 30.9)	2.1	(−1.5 to 4.5)	−15.6	(−33.1 to −3.8)	0.007
	72 h	13.4	(8.8 to 24.4)	1.8	(−2.6 to 4.4)	−11.6	(−25.4 to −4.3)	0.009
Mid-bone of affected bone*	24 h	10.4	(4.7 to 12.7)	1.9	(1.3 to 4.7)	−8.5	(−12.7 to −0.6)	0.05
	72 h	5.0	(−0.3 to 18.8)	0.6	(−4.1 to 2.4)	−4.4	(−20.9 to −1.8)	0.11
Average measurement	24 h	15.0	(11.7 to 21.6)	−2.0	(−3.5 to 1.4)	−17	(−24.7 to −9.9)	0.008
	72 h	14.0	(7.2 to 19.4)	−2.5	(−3.6 to 2.5)	−16.4	(−24.5 to −6.8)	0.0003

^a^Mann–Whitney U test

### Modified glasgow composite pain scores

Three NPWT patients and one control patient had their analgesia reduced prior to scheduled by the analgesia protocol. This was due to sedation and nausea in the presence of a Glasgow composite measure pain score (GCPS) below three in all cases. Two control patients and one NPWT patient had their analgesia increased beyond the protocol. In two cases a fentanyl infusion was required to maintain a GCPS below six during the first 24 h postoperatively while in one control patient, it was not possible to reduce the buprenorphine to 0.02 mg/kg at the scheduled time as the patient became inappetent and scored over six on the GCPS. Buprenorphine reduction in this patient was achieved 24 h later than the standard protocol. The median pain score 24 h postoperatively was three in both the control (IQ: 3 to 4) and NPWT groups (IQ: 1.5 to 4), with no evidence against null hypothesis that the pain scores were equal (Mann–Whitney U test: *p* = 0.57). The median pain score at 48 h postoperatively was three (IQ: 2 to 3) in the control group and one (IQ: 1 to 2.75) in the NPWT group, however there was only very weak evidence to suggest there was a difference between the groups (Mann–Whitney U test: *p* = 0.17). The median pain score at 72 h postoperatively was 1.5 (IQ: 1 to 2.75) in the control group and one (IQ: 1 to 1.75) in the NPWT group, with no evidence to suggest the true difference in ranks was different from 0 (Mann–Whitney U test: *p* = 0.46). The results for the modified GCPS are summarised in Table 3.

**Table 3:** The difference in the median pain scores and discharge scores between control and NPWT groups

		Median (25^th^ to 75^th^ Quartile)				Difference in medians		
Outcome	Time	Control (*n* = 10)		NPWT (*n* = 10)		(95 % CI)		*p*-value^a^
Modified Glasgow Pain Score	24 h	3	(3 to 4)	3	(1.5 to 4)	0	(−2 to 1)	0.57
	48 h	3	(2 to 3)	1	(1 to 2.75)	−2	(−2.5 to 0.5)	0.17
	72 h	1.5	(1 to 2.75)	1	(1 to 1.75)	−0.5	(−2 to 0.5)	0.46
Discharge score	24 h	5	(5 to 5.75)	4	(3.25 to 4.75)	−1	(−2 to 0)	0.009
	72 h	4	(2.25 to 4.75)	2.5	(2 to 3)	−1.5	(−3 to 1)	0.1

^a^Mann-Whitney U test

### Discharge score

The median discharge score was higher in the control group (5, IQ: 5 to 5.75) compared to the NPWT group (4, IQ: 3.25 to 4.75) at 24 h (Mann–Whitney U test: *p* = 0.009), however there was only very weak evidence to suggest a difference between the groups at 72 h (Mann–Whitney U test: *p* = 0.1).

### Dressing changes

Additional dressing changes were required in four (40 %) of controls, however no NPWT patients required additional dressing changes (Fisher’s exact test: *p* = 0.09). Two (20 %) controls and two (20 %) NPWT patients required sedation for dressing changes. The results are summarised in Table 4.

**Table 4:** Differences in dressing changes and complications between control and NPWT groups

	Number (%)		Odds ratio		
Outcome	Control (*n* = 10)^a^	NPWT (*n* = 10)^b^	(95 % CI)		*p*-value^c^
Additional dressing changes required	4 (40 %)	0 (0 %)	0	(0.00 to 1.30)	0.09
Sedation required for dressing changes	2 (20 %)	2 (20 %)	1	(0.06 to 17.08))	1
Short-term complications	4 (40 %)	0 (0 %)	0	(0.00 to 1.30)	0.09
Major long-term complications^d^	3 (37.5 %)	1 (12.5 %)	0.29	(0.00 to 5.49)	0.56
Wound dehiscence	1 (12.5 %)	0 (0 %)	0.0	(0.00 to 34.67)	0.47
Acute infection	0 (0 %)	0 (0 %)	-	-	-
Chronic infection	1 (12.5 %)	3 (37.5 %)	3.84	(0.23 to 250.88)	0.57

^a^There were 8 controls included in the analysis of long-term complications

^b^There were 8 NPWT patients included in the analysis of long-term complications

^c^Fisher’s exact test

^d^Presence of long term complications are unknown for 2 controls and 2 NPWT patients as they were lost-to-follow-up

### Complications

**Fig. 1:**
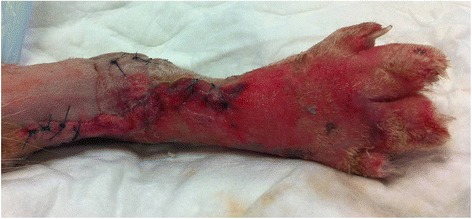
Severe digital swelling 72 h postoperatively following pantarsal arthrodesis using a dorsal plate in a cat in the control group

Short-term complications were seen in four (40 %) of the control group however no short-term complications were observed in NPWT patients (Fisher’s exact test: *p* = 0.09). The complications observed in the control group were all minor and included mild irritation leading to self-trauma, a small area of skin necrosis over incision site, and severe digital swelling 48 to 72 h postoperatively in two cases, one of which received NPWT at 72 h postoperatively (Fig. 1).

Long-term complications were analysed for eight control and eight NPWT patients. Three major long-term complications were seen in the control group (37.5 %) all requiring further surgery; one patient had a screw loosening which necessitated removal of the screw, one patient suffered stress protection of the fracture observed 8 weeks postoperatively which was treated by removing some screws, and one patient had a broken plate. One major long-term complication was seen in the NPWT group (12.5 %) involving multiple screw loosening necessitating revision surgery; this patient also suffered surgical site infection following this second procedure. In terms of minor long-term complications, three patients in the NPWT group (37.5 %) and one patient in the control group (12.5 %) suffered chronic superficial wound infections, all of which resolved with antibiotic therapy. The complications are summarised in Table 4.

When acute wound infection, chronic wound infection and wound dehiscence were analysed separately, there were no acute infections in either the NPWT or control groups; there were three chronic infections in the NPWT group (37.5 %) and one in the control group (12.5 %); there was one case of dehiscence in the control group (12.5 %) and none in the NPWT group.

### Sample size calculations

The a priori sample size for a larger randomised controlled trial was calculated on the basis of the results from the present pilot study. To evaluate whether the increased dehiscence rate in the control group for this study is statistically significant (12.5 % in the control group and 0 % in the NPWT group) at least 31 animals would be required in each group. To evaluate a 25 % difference in the rate of surgical site infection (12.5 % in the control group and 37.5 % in the NPWT group) 45 animals would be needed in each group.

## Discussion

Results from this study demonstrate two clear advantages of using NPWT in comparison to control treatment over these high risk incisions. Postoperative swelling was significantly reduced in patients treated with NPWT and wound discharge was also significantly reduced. This data indicates that use of NPWT may be a viable strategy to reduce wound complications associated with these incisions.

While there is an abundance of information in the human field regarding the use of NPWT on open wounds and a growing body of evidence in the veterinary literature too, the number of studies which have investigated the use of NPWT over closed surgical incisions is low. In the veterinary field, NPWT has been used over free full-thickness meshed skin grafts in dogs and has been shown to expedite granulation tissue formation and reduce graft necrosis [23]. It has also been used to maintain viability of a skin flap in a dog [24]. There are no prior reports in the veterinary literature regarding the routine use of NPWT over closed, high-risk surgical incisions in an attempt to reduce complication rates. Information in the human literature provides promising results in terms of reducing wound dehiscence, infection rates, seroma formation and wound drainage [1, 19, 20, 25] and the results of this preliminary study appear to corroborate these reports in terms of the reduced wound drainage as well as showing a significant decrease in distal limb swelling.

The mechanism of action of NPWT for augmentation of wound healing is not fully understood. Several mechanisms have been proposed including increased blood flow through capillary vessels, oedema reduction and mechanical stretching of cells leading to cell growth and expansion [26, 27]. Postoperative swelling was significantly reduced in the patients in this study where NPWT was used. Potential mechanisms of action which may have contributed to this would include increased microvascular blood flow which has been demonstrated when using NPWT in several previous studies [28–32]. It has been demonstrated that this increased perfusion also occurs when NPWT is applied over intact skin [33] and therefore it seems reasonable to assume that this would also occur over a closed surgical incision. The removal of excess interstitial fluid may contribute to this reduction in swelling. Traumatised tissue, such as in the high-energy fracture patients in this study, may generate excess fluid, as may tissue with compromised vascular outflow such as tissues which are under tension [1]. It has been documented that achieving tension-free closure in arthrodesis cases can be difficult [4] and compromised circulatory drainage of the distal limb is possible [2]. It has been shown that NPWT reduces haematoma and seroma formation in pigs and increases lymph clearance [34]. The incidence and volume of seroma formation following total hip replacements in people is reduced by postoperative NPWT [25]. These methods could certainly have contributed to the reduction in postoperative swelling noted in this study.

The NPWT group had a statistically significant reduction in wound discharge 24 h postoperatively. The use of NPWT has been associated with earlier sealing of wounds [1] and this may partially explain this finding. This earlier sealing of wounds may be related to one of the other mechanisms of action of NPWT; mechanical stress application to the tissue. The application of stress to the surrounding tissues deforms the extracellular matrix and cell cytoskeleton enhancing tissue growth and expansion. This causes the release of intracellular second messengers and up-regulation of immediate early oncogenes associated with cell-growth, proliferation and differentiation [35–37].

While the number of short-term complications and major long-term complications in this study was lower in the NPWT group, this was not statistically significant. When looking at the complications considered particularly relevant to this study (i.e. acute and chronic infection rates and dehiscence rates) the numbers were too small to achieve statistical significance. The number of patients in this initial study is too small to make any conclusions regarding whether this reduced swelling and discharge will yield the desired outcome of decreasing wound dehiscence, infection and general complication rates associated with these incisions. However, the risk of developing a surgical site infection is heavily dependent upon the condition of the wound at the end of the surgical procedure [20]. It is recognised that increased interstitial pressure associated with severe swelling may result in occlusion of the microvasculature and lymphatics which leads to tissue hypoxia and necrosis [1], risk factors for wound infection and dehiscence. In addition, chronic wound interstitial fluid contains elevated levels of collagenases and elastase that degrade extracellular matrix proteins and the resultant degradation products may retard cell adhesion and migration [36]. Therefore the significant reduction in swelling seen in this study could reasonably lead to a reduction in complications overall. A larger scale study would be required to confirm this.

There was only one minor case of wound dehiscence in this pilot study, however, wound dehiscence is a commonly recognised problem following high-energy trauma and arthrodesis [1, 3–5]. It is recognised that the use of sutures and staples induce stress concentrations where they engage the tissue and that elevated stress concentrations can cause ischaemia, fibrosis and tissue injury [21]. This stress concentration can lead to suboptimal contact between the two sides of the incision which can result in haematoma or seroma formation with compromised wound environment, predisposing to complications [21]. Finite element analysis and bench modelling has shown NPWT to reduce the shear stress concentrations at the sutures / staples, reduce lateral stresses around the incision and increase the forces required to disrupt the closed incision [21] and this would be anticipated to reduce the risk of wound dehiscence [38]. The reduction of stress concentrations around the sutures may also reduce the risk of surgical site infection as this will avoid localised areas of ischaemia which reduces the ability to clear infection and can lead to necrosis [39].

It has been proposed that in human patients NPWT over closed surgical incisions may decrease the pain associated with these wounds [22] and therefore GCPS were performed 24, 48 and 72 h postoperatively in this study. There was weak evidence to suggest that the GCPS may be lower in NPWT-treated patients 48 h postoperatively but otherwise no significant differences were found. The significant reduction in swelling discussed above would be expected to be associated with increased comfort in these patients postoperatively and it is possible that the lack of evidence to suggest a reduction in pain is due to the small sample size of the pilot trial. It would be interesting to carry out a larger study to investigate this possibility. While the GCPS has been shown to measure pain [40], assessing pain in animals is inherently difficult and remains subjective, this may have contributed to the lack of significance of these results. This effect could potentially have been reduced by using two or more assessors to score animals at the same time points. In addition, 35 % of the patients in this study were cats, and the GCPS used was not specifically created for use in this species. There are no validated subjective assessment instruments for the evaluation of postoperative pain in cats and therefore the authors considered it to be appropriate to use the GCPS for this study in order to maintain consistency between patients. This is an obvious study limitation however and may have contributed to the lack of significance of these findings. Although the authors adopted a standardised analgesia protocol in this study, unfortunately a relatively large number of patients developed signs which necessitated alteration of this with four patients (20 %) requiring a decrease in opioid analgesia and three (15 %) requiring an increase. This complicates assessment of the effect of closed incision management with NPWT on postoperative discomfort. The authors do not feel that this is a limitation which can ethically be avoided, however, the effects of this would be reduced in a larger scale study.

In this study, 40 % of the control cases required dressing changes over-and-above the scheduled ones at 24 and 72 h postoperatively. No NPWT-treated case required additional dressing changes. While statistical significance was not reached in this small study, this may be a type two error. Reasons for the additional dressing changes in the control patients included strike-through of the dressing and severe digital swelling. Given that both discharge and swelling were statistically significantly reduced in the NPWT-treated patients, it would be reasonable to expect that the number of additional dressing changes would also be less in this group. A larger study would be necessary to confirm this.

One concern of the authors when carrying out this study was that the NPWT-treated patients may have been more likely to require sedation to allow removal of the adhesive sheet associated with the dressing than the control patients. This was not demonstrated in the current study with only two patients in each group requiring sedation for dressing changes, however, with the small numbers examined here, it is possible that this concern may still be shown to be warranted following a larger study.

In this study NPWT was discontinued after the first 24-h postoperative period as detailed in a previous report [22]. To the authors’ knowledge there is no definitive recommendation regarding how long NPWT should be applied to a closed-incision in order to achieve maximal beneficial effect. In a recent review of the use of NPWT for management of incisions following orthopaedic surgery in human patients, nine different studies were evaluated reporting NPWT to be applied for a variable period between 24 h and ten days postoperatively [41]. Further studies are justified in this area to ascertain the optimal duration of NPWT application in both human and veterinary patients.

### Limitations

The authors recognise some significant limitations associated with this study. One of these is the lack of blinding of the investigators. Ideally, the limb measurements, discharge scoring and pain scoring would have been carried out by an individual who was unaware of the dressing which had just been removed. This was primarily not carried out due to the requirement for an additional member of staff to be present at each dressing change for each patient in addition to the primary clinician in charge of the case which was not felt to be logistically possible.

The limb measurement method is also a limitation of the study. The inter- and intratester reliability of morphometric assessment of limb circumference has been shown to be poor in the brachium, crus and thigh, although measurements of the antebrachium gave moderate to fair results [42]. The authors did attempt to maximise the reliability of these measurements by specifying anatomic landmarks and joint positions at which the measurements should be made which have been postulated to be causes of these poor results in previous studies [42]. The authors did not feel that sedation of the patients was warranted in order to achieve more reliable measurements although it has been suggested that this may reduce variation [42]. Reliability of these measurements could have been improved by using a Gulick II tape measure in place of the umbilical tape used here. Umbilical tape was used in this study and disposed of after each use to limit the risk of transmission of nosocomial infection between patients. Another alternative would have been disposable flexible tape measures. The gold-standard in human medicine would be the use of CT or MRI but as these would necessitate sedation of the patient, this was not considered to be a reasonable, ethically justifiable alternative for these patients. In future studies, it would be possible to use CT or MRI for the patients which required sedation for the dressing change regardless, as this would allow a comparison to be made regarding the measurements achieved via these techniques and those achieved using the more routinely adopted morphometric assessment.

The two groups were randomised via envelope pick and the only significant difference in demographic data between the two groups was in age, with the control patients being significantly younger than the NPWT patients. While this is not ideal, this is not felt likely to have adversely affected the results of our study as if anything this would be anticipated to bias the results in favour of control therapy with younger patients being anticipated to heal more uneventfully than older patients as a significant delay in wound healing has been reported in older human patients [43].

The randomisation method chosen in this study resulted in an unequal distribution of cats and dogs in each group as well as an unequal distribution of fracture and arthrodesis cases in each group. This unfortunately resulted in some confounding factors which should be recognised. Differences in first intention healing of sutured wounds have been demonstrated between dogs and cats [44, 45] and interspecies differences have also been reported in the cutaneous vascular supply [46]. Therefore the unequal distribution of cats and dogs in the control and treatment groups may have influenced results. In addition, in the human field, several factors have been shown to influence the incidence of postoperative wound complications following similar interventions including grade of comminution of fracture [47], surgical time [48], delay between injury and surgery [48, 49], wound closure technique [49] and body-mass index of the patient [49]. Variation likely exists in many factors, including some of these mentioned, between high-energy fractures and arthrodesis cases and therefore the unequal distribution of these cases between groups may also have affected results. In future studies, in addition to a larger number of patients, a different randomisation method will be chosen allowing equal distribution of cats and dogs, and fracture and arthrodesis patients between treatment groups. A larger study will also allow the statistical analysis of dogs and cats to be separated in order to allow for further assessment of the interspecies differences in their response to this technique.

## Conclusions

This pilot study demonstrates statistically significant benefits to the use of NPWT over closed, high-risk, surgical incisions in terms of reduced swelling and reduced wound discharge. Following a larger study the authors anticipate that these benefits may result in a reduction in wound-associated complications postoperatively, specifically surgical site infections and wound dehiscence. Further benefits may become apparent following a larger study potentially including decreased levels of discomfort postoperatively and a reduction in the frequency of dressing changes required.

## Methods

This study was a prospective, randomised, controlled pilot study approved by the Ethics and Welfare committee at the Royal Veterinary College (RVC) with the approval number URN 2011 1116. In all cases owners signed a written consent form following a detailed verbal explanation of the study protocol.

### Case selection

Cases were selected from within the population of client owned dogs at the RVC. Recruitment criteria included:

1. Any animal scheduled to undergo either partial- or pan-arthrodesis of the carpus or tarsus for any reason
2. Any animal deemed to have undergone high-energy trauma leading to a distal limb fracture requiring surgical stabilisation. Trauma is difficult to define given that the forces applied to a bone cannot be deduced from a description of the event. For this study, the criteria were considered fulfilled as long as cases with minimal trauma were excluded. The definition of “minimal trauma” in the human literature is “a fall from standing height or less” [50, 51]. Clearly, for quadrupeds this is not strictly transferrable but high energy traumas were considered to be those that were as a result of a motor vehicle incident, a high-height fall or other severe accident where forces encountered were considered likely to be high.

There were several exclusion criteria. Any patient with a coagulopathy, any necrotic tissue in the region of the surgical site or any tumour tissue in the region of the surgical wound was excluded as these are all considered relative or complete contraindications to the use of NPWT [52]. Open fractures, pathologic fractures and low-energy fractures were also excluded.

### Randomisation and case management

Cases were randomised by envelope pick to one of two groups. Group 1 received the current standard of care with a soft support dressing applied postoperatively for 3–5 days; which was changed as necessary during that time. For the purposes of consistent monitoring, during this study the dressing was always changed at 24 h and 72 h postoperatively. Group 2 received NPWT at 125 mmHg for the first 24 h postoperatively in addition to the soft support dressing and then a soft support dressing alone as applied for Group 1. Following the initial 72–120 h study period the requirement for ongoing external coaptation was judged on an individual case basis as determined by the clinician in charge of the case.

Following surgery and postoperative radiography the skin surrounding the wound was cleaned and dried using sterile saline and swabs. For patients randomised to Group 1, a nonadherent absorbent dressing was placed over the wound and a modified Robert Jones dressing applied. For patients randomised to Group 2, the NPWT was applied in standard fashion over the closed surgical incision using a nonadherent absorbent barrier over the closed surgical incision between the skin (and sutures) and the open-cell gauze (Renasys gauze, Smith and Nephew, Kingston upon Hull, England) which was placed over the wound. The transparent adhesive sheet (Renasys adhesive dressing, Smith and Nephew, Kingston upon Hull, England) was then secured to the skin to achieve an air-tight seal. A distance of 3–5 cms circumferential to the wound was covered. This necessitated enclosing the entire distal limb including the foot in smaller patients. A stab incision was made through the adhesive sheet and the adhesive port for the NPWT machine (Renasys port, Smith and Nephew, Kingston upon Hull, England) was applied over this incision. The tubing was positioned such that it coursed proximally toward the trunk. The tubing was attached to the NPWT adjustable pump (Renasys machine, Smith and Nephew, Kingston upon Hull, England) and negative pressure at 125 mmHg was applied continuously. A modified Robert Jones dressing was then applied over the NPWT dressing in the same way as for Group 1 cases.

### Quantitative measurement of swelling

Prior to surgery and whilst under general anaesthesia, for arthrodesis cases the circumference of the limb was measured at the joint proximal to the one to be operated, the joint to be operated, and the joint distal to the joint to be operated. The points on the limb equidistant between these joints were also measured. For distal limb fracture cases the circumference of the limb directly over the fracture site was measured. The circumference of the limb was also measured at the mid-point of this long bone, and at the joints distal and proximal to the fracture. For all cases, the width of the pes / manus was also measured at the level of the proximal interphalangeal joints (PIPJ) and distal interphalangeal joints (DIPJ) in order to allow a measure of digital swelling postoperatively. Similar measurements were taken from the contralateral non-affected limb. Table 5 shows the anatomical landmarks used to maximise consistency between measurements. All joints were measured with the joint held in full extension, other than joints that had been arthrodesed; which were necessarily measured at the angle of arthrodesis. Measurements were made using cotton umbilical tape 0.32 cm in width (Cotton Umbilical Tape, Ethicon, Johnson and Johnson, St-Stevens-Woluwe, Belgium) passed around the limb and marked where the ends met using a permanent marker before measuring the length of the tape using a standard ruler. New umbilical tape was used for each measurement and for each patient to minimise cross-contamination.

**Table 5:** Landmarks used to maximise consistency of quantitative measurement of limb swelling

Joint	Anatomical landmarks
Elbow	Circumferential measurement at level of lateral epicondyle and medial epicondyle with elbow in full extension
Carpus	Circumferential measurement at level of styloid process of radius and styloid process of ulna with carpus in full extension (unless arthrodesis performed)
Metacarpo-phalangeal	Circumferential measurement at the level of the head of metacarpal five laterally and head of metacarpal two medially with joint held in full extension
Proximal interphalangeal	Measurement across dorsum of digits extending from head of proximal phalanx five laterally to head of proximal phalanx two medially with joint held in full extension
Distal interphalangeal	Measurement across dorsum of digits extending from head of middle phalanx five laterally to head of middle phalanx two medially with joint held in full extension
Stifle	Circumferential measurement at level equidistant between the tibial tuberosity and distal extent of the patella with the stifle held in full extension
Tarso-crural	Circumferential measurement at level of lateral malleolus of fibula and medial malleolus of tibia with joint held in full extension
Metatarso-phalangeal	Circumferential measurement at the level of the head of metatarsal five laterally and head of metatarsal two medially with joint held in full extension
Proximal interphalangeal	Measurement across dorsum of digits extending from head of proximal phalanx five laterally to head of proximal phalanx two medially with joint held in full extension
Distal interphalangeal	Measurement across dorsum of digits extending from head of middle phalanx five laterally to head of middle phalanx two medially with joint held in full extension

Limb circumference and paw width measurements were repeated 24 and 72 h postoperatively and at time of last dressing change prior to discharge for all cases and at 48 h intervals for cases remaining in the hospital for longer than 72 h.

The percentage change in limb circumference, measured at the aforementioned six points on the limb that underwent surgery was calculated for measurements conducted at 24 and 72 h post-operatively. The mean of the six measurements was also calculated and compared between control and treatment groups at 24 and 72 h postoperatively. As the middle of the affected bone measurement was not specifically taken from arthrodesis patients, for these cases this was calculated as the average of circumference measurements taken at the middle of the bones proximal and distal to the joint operated.

### Analgesic protocol and pain scoring

A Glasgow Composite Measure Pain Score (GCPS) [40, 53] was completed for all cases every 24 h for the purpose of the study. A standardised analgesia regimen was employed for all patients to allow meaningful comparison. This was altered if health requirements dictated that this was necessary or if a pain score higher than 6/24 or 5/20 was noted. The analgesic protocol differed between dogs and cats. Dogs: All cases received a pure mu (μ) opioid agonist, 0.2 mg/kg methadone intravenously (IV) every 4 h for the first 24 h. Methadone was then decreased to 0.1 mg/kg IV every 4 h for the next 24 h. This was then reduced to a partial μ opioid agonist, buprenorphine IV at 0.02 mg/kg every 6 h for the next 24 h and then 0.01 mg/kg IV every 6 h for the following 24 h. All cases received a non-steroidal anti-inflammatory medication unless a specific contraindication existed. Which specific non-steroidal anti-inflammatory was used varied depending on which drug had been administered prior to presentation. Cats: All cases received a pure mu (μ) opioid agonist 0.2 mg/kg methadone every 6 h IV for the first 24 h. This was then reduced to a partial μ opioid agonist, buprenorphine at 0.02 mg/kg IV every 6 h for the following 48 h before being reduced to 0.01 mg/kg IV every 6 h. All cases received meloxicam 0.05 mg/kg orally every 24 h unless a specific contraindication existed. The differences in the protocol were based upon information in the literature which suggests that buprenorphine is as effective as a pure μ opioid agonist in cats but with a longer duration of action [54–56].

### Qualitative assessment of wound discharge

A wound grade criteria, adapted from that used by Stannard et al. [1], was applied at each dressing change as detailed below.

- Grade 1 – No drainage
- Grade 2 – Scant, no more than 3 small (<4 mm) drops on removed dressing
- Grade 3 – Minimal, 2 or less drops <2 cm in size on removed dressing
- Grade 4 – Mild, spots >2 cm, not full length of incision on removed dressing
- Grade 5 – Moderate, drainage along full length of incision on removed dressing
- Grade 6 – Marked, soaks the dressing between changes

### Additional data

The number of additional dressing changes, in addition to the standardised ones at 24 and 72 h postoperatively, was recorded for each patient. The sedation requirement for the dressing changes was also noted. Outcome data recorded included the development of any complications. Complications were defined as any undesirable outcome associated with the surgical procedure and were classified depending on severity as major (surgical intervention performed) or minor (managed without surgical intervention) and as short-term (occurring during the period of postoperative hospitalisation) or long-term (occurring at anytime thereafter). The development of postoperative infection was also recorded. These were categorised as acute infections (defined as occurring during the initial period of hospitalisation), late infections (defined as occurring at any point following discharge from the hospital) or wound dehiscence. This categorisation was based on previous studies [19]. A wound was considered infected when a purulent discharge, abscess or sinus and / or one or more of the clinical signs of pain and localised swelling, redness, heat, fever or deep incision spontaneous dehiscence was identified on physical examination and / or when an organism was isolated from an aseptically collected sample by culture and / or a positive cytology study [57, 58]. If possible, cases were reassessed at our hospital for suture or staple removal to allow monitoring for wound complications / infection. For those cases unable to return at 10–14 days postoperatively this assessment was performed by the local veterinarian. All cases were scheduled to return for postoperative radiography six to eight weeks postoperatively and where this had not been possible previously, information on wound complications was then collected retrospectively.

### Statistical analysis

The software R, version 2.15.3 (https://www.r-project.org) packages stats, pairwiseCI, boot, epicalc, car and pwr, were used for all calculations. Data are presented as numbers (%) for binary variables or means (95 % CI) for continuous variables where data were normally distributed or median (25^th^ to 75^th^ quartile (IQ)) for non-normal continuous parameters. The analyses were performed on groups as “intention-to-treat”, however as there were no protocol violations in the first 72 h postoperatively, the “per-protocol” results were the same for all results except long-term complications. Demographic data were compared between groups using the Mann–Whitney U test for ordinal data (age and duration of follow-up) or Fisher’s exact test. Differences in postoperative swelling, modified GCPS and discharge scores between the control and NPWT groups were assessed using the Mann–Whitney U tests, with significance level of 5 %. Confidence intervals for the difference in medians were calculated using bootstrap methods. Differences in dressing changes and complications between controls and NPWT patients were assessed using the Fisher’s exact test.

Using the results from the pilot trial, sample size calculations were then conducted to calculate the number of animals which would need to be recruited for a larger study to determine whether NPWT could reduce wound-associated complications postoperatively, specifically surgical site infection and wound dehiscence. The number of animals per treatment group needed to provide 80 % power to detect differences in wound dehiscence rate and the rates of infection found in the pilot study at a significance level of 5 % was calculated.

## Abbreviations

DIPJ: Distal Interphalangeal Joint
GCPS: Glasgow Composite Measure Pain Score
IV: Intravenous
NPWT: Negative Pressure Wound Therapy
PIPJ: Proximal Interphalangeal Joint
RVC: Royal Veterinary College
